# Etiologies of diarrhea and drug susceptibility patterns of bacterial isolates among under-five year children in refugee camps in Gambella Region, Ethiopia: a case control study

**DOI:** 10.1186/s12879-019-4599-6

**Published:** 2019-11-28

**Authors:** Getachew Kabew Mekonnen, Bezatu Mengistie, Geremew Sahilu, Helmut Kloos, Worku Mulat

**Affiliations:** 1 Addis Ababa University, Ethiopian Institute of Water Resources, PO. BOX 150461, Addis Ababa, Ethiopia; 2 Addis Ababa City Administration, PO. Box 8470, Addis Ababa, Ethiopia; 3 Haramaya University, College of Health and Medical Sciences, PO. Box 1570, Harar, Ethiopia; 4 Department of Epidemiology and Biostatistics, University of California, San Francisco, USA; 5 Wello University, College of Medicine and Health Sciences, Desse, Ethiopia

**Keywords:** Diarrhea, Under-five children, Etiologies, Drug susceptibility, Refugees

## Abstract

**Background:**

Despite substantial global effort and updated clinical management guidelines, diarrhea continues to be among leading worldwide causes of morbidity and mortality in children. Infectious diarrhea, the most common form of diarrhea causes substantial morbidity and mortality among children in developing countries, and the muddled use of antibiotics needs caution due to potential problems of drug-resistance. The aim of this study is to identify etiologies of diarrhea and drug susceptibility patterns of bacterial isolates in under-five children in refugee camps in Gambella Region, Ethiopia.

**Methods:**

An institution- based matched case control study was conducted using a questionnaire-based interview from June to December 2017 in Pugnido and Teirkidi refugee camps. Stool samples were collected and parasites causing diarrhea were identified by wet mount microscopy. Conventional culture supplemented with API 20E identification kit was used to identify *Salmonella* and *Shigella* species. Antibiotic susceptibility of bacterial isolates was investigated by using the disk diffusion method. The association between etiologies and diarrhea was analyzed using McNemar test or Fisher exact test with 95% confidence interval at a level of significance of *P < 0.05*.

**Results:**

The overall prevalence of enteric pathogens were 55 (41.0%) in diarrhea cases and 18 (13.4%) in healthy controls. The detected etiologies include *Giardia lambia* (28), *Shigella* spp. (16), E. *hystolyotica/dispar* (13), *Ascaris lumbricoides* (10), *Salmonella* spp. (6), *Cryptosporidium parvum* (6), *Hymenolepis nana* (4) and *Isospora belli* (3). All isolates were sensitive to kanamycine and ceftazidime. The high resistance rate was observed against ampicillin (100%), amoxicillin (100%), erythromycin (52%), chloramphenicol (47.5%), tetracycline (40.5%), cotrimoxazole (34.8%) and amoxicillin-clavulanic acid (33%). The majorities of the isolates had a low rate of resistance to ciprofloxacin (8.7%), naldxic acid (8.7%) and amikacin (13%).

**Conclusions:**

*Giardia lamblia*, *E. Hystolytica*/dispar, and *Shigella spp* are the common etiologies of diarrhea in children in the studied refugee camps*.* The study also showed that significant numbers of bacterial isolates were resistant to the commonly used antimicrobial drugs. Therefore, improving clinical laboratory services and promoting evidence-based drug prescription may reinforce proper use of antibiotics and reduce the emergence of microbial resistance.

## Background

Despite substantial global effort and updated clinical management guidelines, diarrhea continues to be among the leading causes of morbidity and mortality in human worldwide, accounting for around 2.5 billion cases and 2.4 million deaths each year [1]. Diarrhea comprises more than 25% of all deaths among refugees [2]. Children under five are particularly vulnerable [3] and episodes of diarrhea account for about 28% of all hospital visits for this age group [4]. Children in refugee camps tend to be under stress have poorer nutritional status and are more susceptible to severe diarrhea and dehydration than non-refugee children [5]. Drinking water contaminated by human and animal feces contributes significantly to diarrheal disease [6]. Diarrhea also spreads through contaminated food via the fecal-oral route, by house flies or person-to-person transmission as a result of poor hygiene [7]. The heaviest diarrhea burden exists in rural African communities, where most people are living with inadequate water, sanitation and hygiene and health care services [8]. Infectious diseases such as diarrhea usually result in higher rates of morbidity and mortality in malnourished people. Nearly, 31% of all children under-5 years of age in developing countries are underweight, 38% experience stunted growth and 9% show wasting [9].

Diarrhea can be caused by both infectious and non-infectious agents [10]. Severity of the illness usually depends to some extent on the etiologies and child age [11]. Globally, infectious diarrhea that may be caused by viruses, bacteria or protozoa [12]. The leading and vaccine preventable cause of severe diarrhea among children under five is rotavirus [13]. Non-typhoid *Salmonella*, *Shigella* species, *Salmonella typhi*, enterotoxigenic *Escherichia coli* (ETEC), *Campylobacter jejuni* and *Vibrio cholerae* account for most diarrheal cases in the developing world [14]. Sever forms of diarrhea are also obsereved in *Cryptosporidium parvum*, *Giardia lamblia* and *Entamoeba histolytica* infections. Combined administration of oral rehydration salt (ORS) and zinc has been reported to alleviate diarrheal symptoms and expedite recovery of many patients in different parts of the world [15]. Antimicrobial therapy shortens the duration of the illness, prevents development of complications and reduces the severity of associated symptoms such as fever and abdominal pain [16]. It also decreases subordinate cases by person-to-person spread of diarrhea pathogens. While prescribing antibiotics for the treatment of diarrhea, clinicians have not only to be aware of the most likely pathogens, but also of their antimicrobial susceptibility patterns and safety profiles [16].

The health care systems used in the treatment of diarrhea in the developing world are poorly organized and virtually unregulated [17]. Early diagnosis and treatment of diarrhea is hampered by lack of laboratory facilities in remote areas, where diarrheal disease is most prevalent and is a major cause of child mortality [18]. In rural Ethiopia, clinical investigations of diarrheal diseases are usually restricted to a conventional microscopic examination of stool samples for the identification of helminth eggs and protozoan trophozoites and cysts [19]. Antibiotic treatment is recommended mainly for cases with acute bloody diarrhea in children [16]. However, the muddled use of antibiotics in the developing world needs caution due to potential problems of drug-resistance, side-effects and cost of treatment [20]**.** The scarcity of data on the enteric pathogens profile of refugee populations in Sub-Saharan Africa in general and Ethiopia in particular urges for studies that facilitate the development of effective treatment strategies and diarrhea prevention efforts. Therefore, this study was conducted to investigate etiologies of diarrhea and drug susceptibility patterns of bacterial isolates among under-five children visiting health centers in the refugee camps in refugee camps in Gambella Region, Ethiopia. Furthermore, we assessed the association between malnutrition and diarrhea in under-five children.

## Methods

### Study sites

The Gambella Regional State is one of the 11 administrative regions of Ethiopia located in western Ethiopia at the border with south Sudan. Gambella has 14 *weredas* (districts) and borders with the Oromiya Regional State to the north and east and the Southern Nations, Nationalities and Peoples’ Regional State (SNNPRS) to the south. Ethiopia is hosting the second largest refugee population in Africa, sheltering 889,071 registered refugees and asylum seekers as of October 2015. South Sudanese refugees who are scattered in various locations across western Ethiopia accounted for **411,366 (**46%) of the total [21]. Pugnido and Teirkidi refugee camps were randomly selected among Gewi, Kule and Nguenyyiel camps in Gambella Region. A total of 12,553 children under age five are living in Pungnido and 10, 150 in Teirkidi refugee camps.

### Setting and enrollment

An institution- based matched case control study was conducted to identify common etiologies of diarrhea from June 2017 to December 2017 in the two health centers in Pugnido and Teirkidi refugee camps. The capacity of clinical laboraloties in health centers in the refugee camps was limited to wet mount microscopy in examining stool samples. The study enrolled diarrheic children less than 5 years of age who visited the health centers in the study period. We defined acute diarrhea as abrupt onset of loose or watery stools at least three times in a 24-h period within the 14 days preceding the survey [22]. Irrespective of frequency, blood in stool or dysentery also indicates acute diarrheal illness [23]. Chronic or persistent diarrhea episodes begin acutely but last for more than 14 days [22]. Clinicians checked for the symptoms commonly accompanied by diarrhea, such as cramps, fever, and abdominal pain and vomiting. A control was selected for each case recruited and they were individually matched for their sex (one to one matching). Controls were non-diarrheic children attending the pediatric clinics for a check-up or routine immunization and who did not receive any antibiotic 2 weeks prior to the study.

### Exclusion criteria

Study subjects who took antibiotic within the previous 2 weeks or who were critically ill and unable to provide a stool sample were excluded from the case control study. Asymptomatic controls who had a history of diarrhea during the previous 2 weeks. Subjucts those who had been diagnosed positive for immune compromising diseases, including HIV/AIDS, were also excluded from the study.

### Sample size

The sample size was calculated based on a similar study conducted in 2013, Kenya on the etiology and factors associated with bacterial diarrheal diseases among urban refugee children, which reported 17.0% for diarrheic cases and 2.4% for controls [24]. Therefore: considering, Z_1-β_ = 0.84 (power of 80%), 95% confidence interval, 2 design effect with the proportion of case and control is assumed to be 1:1 with a 10% non-response rate, the sample is estimated using the case control sample size calculator tool of Open Epi software. Thus, the total sample size (both groups) was 268, 134 cases and controls each. P1 = proportion of diarrheic children exposed to bacterial isolates, the p2 = proportion of non-diarrheic children exposed to pathogenic bacteria.

### Sampling and data collection

Interviews were conducted using the annexed tool (Additional file 1) on the first day of patient visiting of the refugee’s clinic in the native language (Agnuak or Nuer) of the respondents regarding sociodemographic characteristics, antecedent exposure and clinical information such as stool frequency, level of dehydration, malnutrition status, diarrheal duration and treatment before the health center visit using clinical information sheets.

At enrollment, a nurse measured the child’s weight (in kilograms) and height (in centimeters) to determine their nutritional status using the new World Health Organization (WHO) child growth standards 2006 [25] and its association with enteric pathogens. The height of infant age 6–23 months measured in recumbent position while for children age 24–59 months was measured in standing position. The Weight of the child was measured to the nearest 10 g by UNICEF electronic scale. For a child not stand alone the mother was weighted together with a child and then without the child. The difference between the two measurements was taken as the child’s weight. The levels of stunting (height for age z-scores), underweight (weight for age z-scores), and wasting (weight for height z-score) were calculated using Antro Plus software. Thus, children who were below − 2 standard deviations for height for age, weight for age, and weight for height were defined as stunted, underweight, and wasted, respectively. Wasting indicates recent weight loss, whereas stunting usually results from being chronically underweight. The degree of dehydration was also assessed and recognized as mild, moderate or severe according to WHO clinical guidelines [22].

Each case and control submitted at least 3 g fresh stool sample. Within 30 min of passage a portion of each fecal sample was examined for the presence of protozoan parasites using wet mount direct microscopic method and thin smears were prepared, air dried, stained with modified acid fast stain and examined for *Isospora belli* and *Cryptosporidium parvum*. The use of microscopy does not allow us to differentiate *E. histolytica* from the morphologically similar *E. dispar* [26]. The second portion were inoculated into two carry-Blair transport media and placed in cold storage (4 °C) [27] in with triple packages. Then it was transferred to the microbiology laboratory of Gambella Regional Public Health Institute within 48 h for bacterial identification and drug susceptibility tests.

Enteric bacterial pathogens were investigated using conventional culture methods and the API 20E kit. A loop full of swab from Cary-Blair tubes were inoculated aseptically on MacConkey (Oxoid) for isolation of *Salmonella* and *Shigella*. For *Salmonella* enrichment, samples were also inoculated in Selenite-F broth (Oxoid), and then sub-cultured on S-S agar. The culture media were incubated at 37 °C for 24 h and the plates examined for growth of lactose and non-lactose fermenting colonies [28]. These were further identified using API 20E kits, a reliable means to identify members of the family Enterobacteriaceae after overnight incubation [29]. Isolates were identified according to their biochemical behavior in the conventional tests [30], and using Apiweb for the interpretation of the API 20E test results.

Antibiotic susceptibility of bacterial isolates was determined using the disk diffusion method using Muller Hinton agar as described by the National Committee for Clinical Laboratory Standard [30]. The panel of the 14 antimicrobials tested was amoxicillin (AMX, 25 μg), amoxicillin-clavulanic acid (AMC, 20/10 μg), ampicillin (AMP, 10 μg), cefotaxime (CTX, 30 μg), ceftazidime (CAZ, 30 μg), chloramphenicol (CN, 30 μg), Tetracycline (TTC, 30 μg), kanamycin KA, 30 μg), nalidixic acid (NA, 30 μg), ciprofloxacin (CIP, 5 μg), co-trimoxazole (trimethoprim-sulfamethoxazole, SXT, 1,25/23,75 μg), gentamicin (GM, 15 μg), amikacin (AN, 30 μg), and erythromycin (E, 30 μg)`.

### Quality assurance

In order to assure the quality of the data, a structured questionnaire was developed to the context of the study area and culture by reviewing literatures from similar studies. The instrument was translated into Agnwak and Nuer languages to make it clearer for the respondents. Then it was re-translated into English to check the consistency of each question. Data collectors were experienced nurse or health officer professionals who have been working in the health centers. Training was given to the data collectors a week prior to the study to ensure that the principles of human subjects research and of good clinical practices were adhered to [31], how to conduct interviews in regard to issues of privacy, asking questions in a nonjudgmental way, and building rapport with the respondents, how to perform a focused physical examination, and how to collect, process, and transport stool specimens. Pre-tests were conducted to ensure clarity of wording, logical sequence and to maintain pattern of the questions, to avoid information distortion. A few changes were then made in the questionnaire. Stool sample collection and storage complied with a standard protocol which should be applied in enteric pathogen investigating laboratories. Control strains *Escherichia coli* ATCC 25922, *Salmonella typhimurium* ATCC 14028 and negative controls (sterile distilled water) were used to ensure the quality of the media and testing breakpoints for MIC of antibiotics [32]. Quality reagents and standard operational procedures acquired from recognized manufacturers were used and followed.

### Data analysis

The data were double- entered using Stata version 13.1, reviewed and checked for completeness, cleanness and consistency. Descriptive statistics (frequencies, proportion) were compared using a chi- square test. The results are presented using tables, pie charts and bar graphs. We examined the association between diarrhea and impaired growth, using *Z* scores for weight- for- age (undernutrition), height- for- age (stunting), and weight -for- height (wasting) as described previously [33]. The association between diarrhea and etiologies was determined by using the McNemar test or Fisher exact test. Statistical tests were performed with a level of significance of P - value 0.05.

## Results

### Characteristics of cases and controls

A total of 134 paired diarrheic and asymptomatic children were included in this study. Of these, 64 and 70 pair of subjects were involved in health centers of Pugnido and Terikidi refugee camps, respectively. One hundred forty-eight (55.2%) of the study participants were females and 120 (44.8%) were males with a mean age of 21.3 (range 1 to 58) months. Seventy (26.1%) of the child birth order was 2nd, followed by 54 (20.1%) 3rd ordered, 49 (18.3%) 4th ordered, 48 (17.9%) 1st ordered and 47 (17.5%) 5th and above ordered children. More than 95% (257) of the caregivers were female. The majority (90.7%) of the interviewed caregivers were mothers followed by sisters (4.9%), fathers (2.6%), other relatives (1.1%) and brothers (0.7%). Eighty (30%) of the caregivers were Agnuak while 188 (70.1%) of them were Nuer. All of the caregivers were South Sudanese and their mean age was 27.0 (range 15 to 56) years. Two hundred six (76.9%) caregivers had not attended formal education and 227 (84.7%) of them were married (Table 1).

**Table 1:** Characteristics of diarrhea cases and controls in children in Pugnido and Teirkidi refugee camps, Gambella Region, Ethiopia, 2017

Variable	Cases	Controls
Child sex		
Female	74 (55.2)	74 (55.2)
Male	60 (44.8)	60 (44.8)
Birth order of the child		
1st	27 (20.1)	21 (15.7)
2nd	31 (23.1)	39 (29.1)
3rd	28 (20.9)	26 (19.4)
4th	28 (20.9)	21(15.7)
5th and above	20 (14.9)	27 (20.1)
Caregivers’ age category (years), N (%)		
< 25	56 (41.8)	29 (21.6)
25–34	58 (43.3)	82 (61.2)
≥ 35	20 (14.9)	23 (17.2)
Caregivers’ sex, N (%)		
Male	4 (3)	7 (5.2)
Female	130 (97)	127 (94.8)
Caregivers’ marital status, N (%)		
Single	6 (4.5)	6 (4.5)
Married	117 (87.3)	110 (82.1)
Divorced	7 (5.2)	6 (4.5)
Widowed	4 (3)	12 (9)
Ethnicity of caregivers		
Agnuak	40 (30)	40 (30)
Nuer	94 (70)	94 (70)
Caregiver educational level		
No formal education	96 (71.6)	110 (82.1)
Primary school (1 to 8th grade)	25 (18.7)	16 (11.9)
Secondary (9 to 12th grade)	9 (6.7)	8 (6.0)
Diploma and above	4 (3.0)	0 (0)
Child mean age in months (SD)	20.3 (13.3)	22.3 (15.1)
Child mean weight in kg (SD)	9.2 (2.3)	9.7 (2.6)
Child mean height in cm (SD)	82.8 (15.0)	82.7 (20.2)
Positive stool samples, No (%)	56 (41.8)	17 (12.7)

Of 134 children with diarrhea, 109 (81.3%) experienced vomiting, and 97 (72.4%) of fever and 55 (41%) of abdominal pain. The mean duration of diarrhea among cases was 3.8 days and the frequency of diarrhea was 4.3 episodes within the 24 h preceding the survey. Danger signs of diarrhea were observed in 35 (26.1%) of the cases (Table 2).

**Table 2:** Clinical information about diarrhea cases among children in Pugnido and Teirkdi refugee camps in Gambella Region, Ethiopia

Clinical information about the cases, (*n* = 134)	Frequency (%)
Diarrhea onset	
< 14 days	128 (95.5)
≥ 14 days	6 (4.5)
Frequency of diarrhea within the previous 24 h	
3–5	111 (82.8)
≥ 6	23 (17.2)
Treatment before attending the health center	
Yes	14 (10.5)
No	119 (89.5)
Danger signs observed	
Yes	35 (26.1)
No	99 (73.9)
Number of cases with the danger sign (*n* = 35)	
Blood in stool	12 (34.3)
Thrist/dry mouth	20 (57.1)
Sunken eyeballs	8 (22.9)
Tearless eye	3 (8.6)
Loss of the stretchiness of the skin	5 (14.3)
Treatment at the health facility	
Yes	115 (85.8)
No	19 (14.2)
Treatment type given at the health facility (*n* = 115)	
ORS	101 (87.8)
Antibiotic	75 (65.2)
Zinc	31 (27.0)
Anti-parasitic drug	19 (16.5)
IV fluid	5 (4.4)
Dehydration level	
Some (mild or moderate)	14 (5.2)
Severe	3 (1.1)
None	251 (93.7)

### Etiologies among cases and controls

**Fig. 1:**
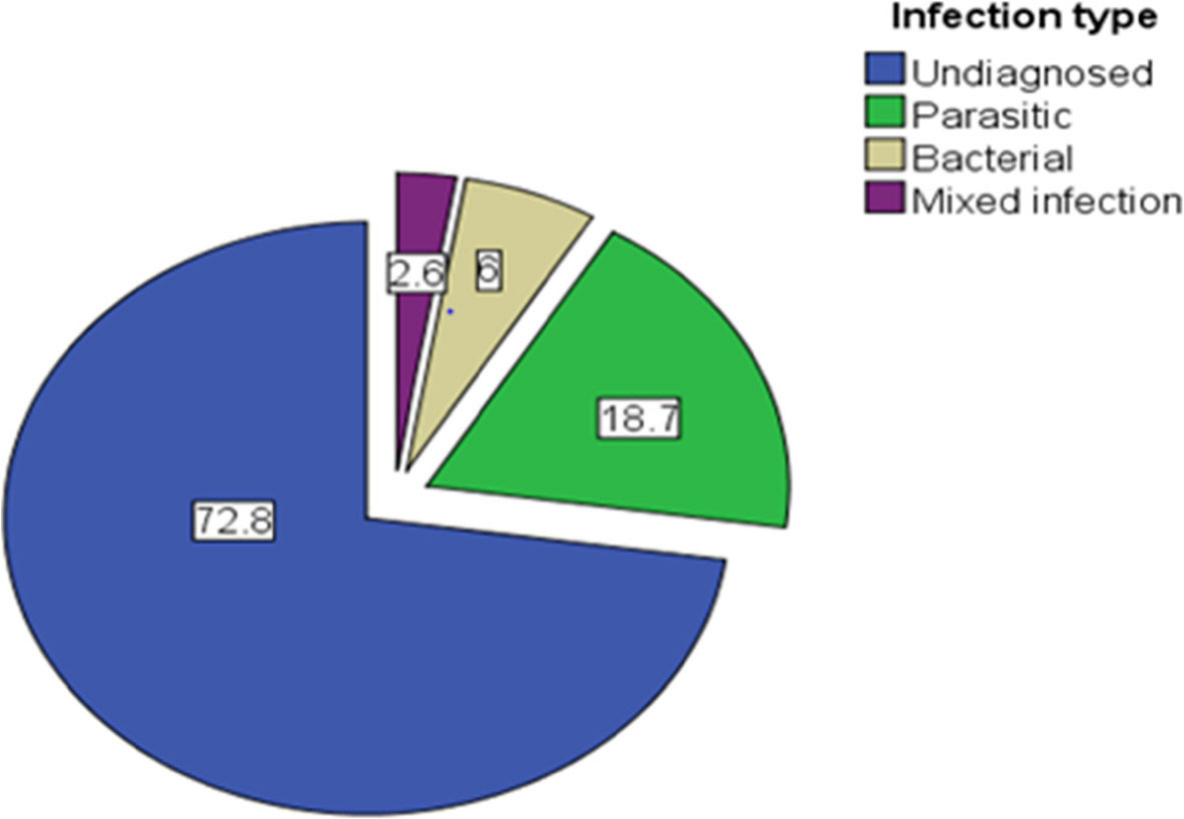
Percent of enteric infections detected from under-five children visited the health centers at refugee camps in Gambella Region, Ethiopia in 2017

Stool samples were collected from the 134 consecutive cases and 134 controls in the two health centers. Subjects who failed to give a stool sample were immediately substituted by another eligible case or control to attain the required sample size. A total of 86 enteric pathogens was detected from the 73 (27.2%) positive stool samples. The overall prevalence of enteric pathogens in diarrhea cases and healthy controls was 56 (*P* = 41.8, 95% CI 0.34 0.5) and 17 (*P* = 12.7, 95% CI 0.08 0.195), respectively. Bacterial and parasitic infections comprised 6 and 18.7%, respectively. Mixed infection was detected in 2.6% (Fig. 1).

More than one-third, 26 (35.6%) of the enteric pathogens were detected in age group between 12 to 23 months. The detected etiologies include *Giardia lamblia* (28), *Shigella spp.,* (16), *E. hystolytica/dispar* (13), Ascaris *lumbricoides* (10), *Salmonella spp.* (6), *Cryptosporidium parvum* (6), *Hymenolepis nana* (4) and *Isospora belli* (3). A significant difference was observed between cases and controls in the detection of *Giardia lamblia*, *Shigella spp.,* and *E. hystolytica/dispar.* The prevalence of stunting, wasting and underweight among study participants were 33.2, 22.8 and 29.5%, respectively. There were statistically significant differences between cases and controls in wasting and underweight parameters (Table 3).

**Table 3:** Frequencies of detection of etiological agents causing diarrhea and nutritional status in children at refugee camps in Gambella Region, Ethiopia

Variable	Frequency (%) detection		*P* Value
	Cases (*n* = 134)	Control (*n* = 134)	
Enteric Pathogen detected			
*Giardia lamblia*	21 (15.7)	7 (5.2)	0.008*
*E. hystolytica/dispar*	11 (8.2)	2 (1.5)	0.013*
*Ascaris lumbricoides*	5 (3.7)	4 (3.0)	0.74
*Cryptosporidium parvum*	5 (3.7)	1 (0.7)	0.1
*Hymenolepis nana*	3 (2.2)	1 (0.7)	0.32
*Isosphora belli*	2 (1.5)	1 (0.7)	0.56
*Shigella species*	14 (10.5)	2 (1.5)	0.003*
*Salmonella species*	4 (3.0)	2 (1.49)	0.26
Co-infection	10 (7.5)	2 (1.5)	0.034*
Nutritional status			
Stunted			
Yes	51 (38.1)	38 (28.4)	0.12
No	83 (61.9)	96 (71.6)	
Wasted			
Yes	39 (29.1)	22 (16.4)	0.019*
No	95 (70.9)	112 (83.6)	
Underweight			
Yes	50 (37.3)	29 (21.6)	0.07*
No	84 (62.7)	105 (78.4)	

* Indicates that there was a significant difference between diarrhea cases and controls at *p* < 0.05

### Antimicrobial susceptibility testing

**Fig. 2:**
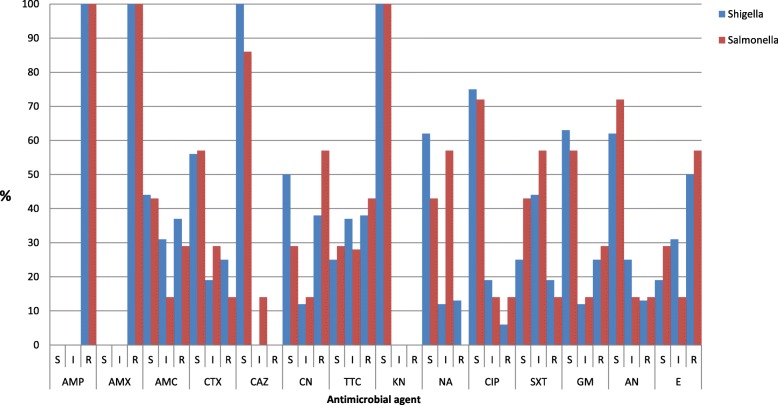
Antimicrobial susceptibility patterns of salmonella and shigella isolates from stool samples of under-five children in refugee camps in Gambella Region, Ethiopia in 2017

All the isolates were sensitive to kanamycine and ceftazidime but were entirely resistant to ampicillin (100%) and amoxicillin (100%). High rates of resistance were also observed against erythromycin (52%), chloramphenicol (47.5%), tetracycline (40.5%), cotrimoxazole (34.8%) and amoxicillin-clavulanic acid (33%). The majority of the isolates had low rates of resistance to ciprofloxacin (8.7%), naldxic acid (8.7%) and amikacin (13%). Resistance to more than two antibiotics was observed among most (87%) isolated enteric pathogens (Fig. 2).

## Discussions

A significantly higher rate of enteric pathogens was detected from the diarrhea cases compared to the controls. However, the rates of detections in the present study are lower than those reported by studies in Egypt, Jordan and Denmark [34–36]. The reason could be that the range of detected pathogens varied due to differences in microbiological methods used. Birth order of the child had significant association with infectious diarrhea in some studies [37]. However, it was not observed in our study. Prevalence of enteric parasites were higher among cases (32.1%) than controls (10.4%), comparable with other studies, showing that parasitic infections remain the common cause of childhood diarrhea in Gambella region [38]. Corresponding to other studies [39] *salmonella* and *shigella* species were identified from 18 (13.5%) diarrhea cases but in only 4 (3.0%) controls. The prevalence of co-infection with more than one enteric pathogens were significantly higher in cases than in healthy controls which is in line with similar studies [40]. The synergy ill effect of enteric pathogens in humans has been repeatedly described [41].

Statistical significance was observed between cases and controls for *Giardia lamblia, E .hystolytica/dispar,* and *Shigella* spp. While there was no difference for *Ascaris lumbricoides*, *Cryptosporidium parvum, Salmonella spp*, *Hymenolepis nana* and *Isospora belli*. These results confirmed that *Giardia lamblia, E .hystolytica/dispar* and *Shigella* spp. were agents associated with diarrhea among children in the study area. These findings are in line with most studies [14] done in rural areas vulnerable to diarrhea and are mainly attributable to inadequate water, sanitation and hygiene (WASH) services. The study also showed that diarrhea was significantly associated with wasting and underweight, supporting the fact that malnutrition is one of the factor commonly linked to diarrhea [9]. However, stunting was a shared malnutrition problem by both the studied cases and controls.

All *Shigella spp.* and *Salmonella spp.* were fully resistant to amoxicillin and ampicillin. High rates of resistance were also observed to erythromycin (52.0%), chloramphenicol (47.5%) and tetracycline (40.5%). These findings are in line with other studies conducted in Ethiopia [39], indicating that muddled and less diversified uses of antibiotics may lead to emergence of resistant strains [42]. On the other hand, isolates were entirely susceptible to kanamycin and ceftazidime, which are recently, introduced new drugs in Ethiopia. The isolates also showed that high rate of susceptibility to naldxic acid (91.3%), ciprofloxacin (91.3%), amikacin (87%) and cefotaxime (78.3%). Drug resistance to more than two antibiotics was observed in the majority (86.9%) of the isolated enteric bacterial pathogens unlike in other studies [43]. This might be due to the high rate of drug resistance or use of larger numbers of antibiotics tested in the study area. *Salmonella* isolates’ are more resistant to chloramphenicol and ciprofloxacin than *shigella* isolates. This could be due to the rampant antimicrobial use in animal production systems suspected to be a cause of the emergence and dissemination of a conjugative plasmid antimicrobial resistant gene of salmonella [44].

### Strength and limitations of the study

This study is the first study explored the common etiologies of childhood diarrhea at refugee camps in one of the remote Region in Ethiopia, where skilled manpower and laboratory facilities are very scarce. For financial reasons the study did not include viral tests and unable to identify some bacterial enteric pathogens. The study also did not determine the seasonal variety of enteric infections.

## Conclusions

This study indicates that the parasites *Giardia lamblia,* and *E. hystolytica*/*dispar* and *Shigella species* are the common isolates among individuals with diarrhea whose stool has been tested*.* Malnutrition had an important association with diarrhea morbidity. The study also showed that rate of antimicrobial resistance was extremely high among *shigella* and *salmonella* species. All *Shigella and Salmonella isolates were* resistant to Amoxicillin and ampicillin. Therefore, improving clinical laboratory services and promoting evidence- based drug prescription may reinforce the proper use of antibiotics and reduce the emergence of microbial resistance. A prospective study that covers the potential viral and bacterial enteric pathogens and their seasonal variability is needed to shed further information on the dynamics of infectious diarrhea in the refugee camps.

## Supplementary information

**Additional file 1.** ANNEX VII Assessment Tools for A Case/Control study.

## Data Availability

The relevant data supporting this publication are summarized with tables in the manuscript. However, the raw data can be accessed from the principal author (GK) whenever required using appropriate procedures and format.

## Abbreviations

AAU: Addis Ababa University
ARRA: Administration for Refugees and Returnees Affairs
EIWR: Ethiopian Institute of Water Resources
UNHCR: United Nations High Commissioner for Refugees
WASH: Water, Sanitation and Hygiene
WHO: World Health Organization
