# Supplementary material for: Etiologies of diarrhea and drug susceptibility patterns of bacterial isolates among under-five year children in refugee camps in Gambella Region, Ethiopia: a case control study
Source: BMC Infect Dis. 2019 Nov 28;19:1008. doi: 10.1186/s12879-019-4599-6 (PMC6883563; doi:10.1186/s12879-019-4599-6)
Supplement: Supplementary file 1 — Additional file 1. ANNEX VII Assessment Tools for A Case/Control study. [file 12879_2019_4599_MOESM1_ESM.docx]

ANNEX VII **Assessment Tools for A Case/Control study**

**A.** **Interview** **Questionnaire**

**Unique code number: ________ Patient Card number: ________**

**Refuge camp** ______________________ **Heath facilite Name** _________________

Case/Control matching code no (similar between a case and its control) ____________

Name of interviewer _________________________Signature ______ Date_________

| QNo | Question | Response |
| --- | --- | --- |
| 101 | What is the child Age? | _______in completed month |
| 102 | Child sex | 1. Male 2. Female |
| 103 | Birth order of a child | 1. 1^st^ 2. 2^nd^ 3. 3^rd^ 4. 4^th^ 5. 5^th^ 6. Other _____ |
| 104 | Your relation with the Child | 1. Mother 2. Father 3. Sister 4. Brother   5. Other (specify) ___________ |
| 105 | Sex of the caregiver | 1. Male 2. Female |
| 106 | Age of the caregiver | ________ in full year |
| 107 | What is your marital status? | 1. Single 2. Married 3. Divorced 4. Widowed 5. Other |
| 108 | What is your Religion? | 1. Muslim 2. Orthodox 3. Protestant 4. Catholic 5. Animism 2. Others________ |
| 109 | What is your nationality? | 1. South Sudanese 2. Sudanese 3. Kenyan 4. Eritrean   5. Other |
| 110 | What is your Ethnicity? | 1. Agnuak 2. Nuer 3. Dinka 4. Madi 5. [Acholi](https://en.wikipedia.org/wiki/Category:Acholi)‎   6. Others_________ |
| 111 | Where was your residence before? | 1. Urban 2. Rural |
| 112 | What was your occupation before displacement? | 1. Farmer 2. Housewife 3. Merchant 4. Government employee 5. Student 6. Commercial sex worker 7. Daily laborer 8. Unemployed 9. Others-------------- |
| 113 | What is your highest educational level completed? | 1. Unable to read and write 2. Primary school(1- 8^th^ grade) 3. Secondary school(9-12^th^ grade) 4. Diploma and above(12^th^ +) 5. Informal education(able to write and read) |
| 114 | For how long you have been here in the refugee camp with the child? | _________ |
| 115 | Do both parents live with the child here? | 1. Yes 2. No   If not, specify which of the parent is absent_________ |
| 116 | How many people live in your house on a regular basis? | 1. Males ____ 2. Females ____   Total _______ |
| 117 | What is the number of household members in each age category? | 1. Less than five years ______ 2. 5 – 15 years ________ 3. 16 – 30 years___________ 4. 31 – 65 years __________ 5. > 65 years____________ |

**B.** **Clinical Data Check List**

**Unique code number:** ________ **Patient Card number:** ________

**Patient unique code** ________ **Name of the health center** ________

| **Clinical assessment** | **Finding/Judgment** | **Remark** |
| --- | --- | --- |
| Child patient type | 1. Inpatient/Admitted 2. Outpatient 3. Other _______ |  |
| Number of days with diarrhea (for diarrhea cases only) | ___________ |  |
| Stool frequency per day/last 24 hours (for diarrhea cases only) | ________ |  |
| Has the child vomiting?  If yes, vomiting frequency per day | 1. Yes 2. No   _________ |  |
| Has the child fever? | 1. Yes 2. No |  |
| Has the child abdominal pain? | 1. Yes 2. No |  |
| **Anthropometric measurements of the child**  Weight in Kg  Height in cm  Mid Upper Arm Circumference (MUAC) in cm  Weight-for-height (z score)  Height-for-age (z score)  Weight-for-age (z score) | ________  ________  ________  ________  _________  _________ |  |
| Is there any danger signs observed? | 1. Yes 2. No |  |
| If yes, for the above question, which danger signs of diarrhea seen? | 1. Passage of >3 loose stools with blood in 24 hours 2. Thirst and dry mouth 3. Sunken eyeballs 4. Tearless eyes 5. Loss of stretchiness of the skin 6. Others ___________ |  |
| Degree of malnutrition | 1. None 2. Mild 3. Severe |  |
| Types of malnutrition | 1. Marasmus 2. Kwashiorkor  3. Marasmic-kwashiorkor 4. Other |  |
| Patient’s dehydration status: | 1. None 2. Some 4. Sever |  |
| Did the child contact to any diarrheal patient in the last 7 days? | 1. Yes 2. No |  |
| Has the child been treated with antibiotics within the last two weeks?  If yes, so when/why/ name of the drug? | 1. Yes 2. No   _________ __________  __________ |  |
| Does the patient require treatment now? | 1. Yes 2. No |  |
| If yes, what type of treatment administered in clinic/hospital visit (for diarrhea cases only): | 1. Oral rehydration solution intake 2. Antibiotics ________________ 3. Zinc 4. Anti-parasitic ___________ 5. IV fluid_______ 6. Others _______ |  |
| Follow up needed (for diarrhea cases only) | 1. Yes 2. No 3. Not determined |  |
| Stool investigation request date (for both groups): | _____/_____/_______ |  |
| Clinician/examiner name and signature | ___________/___________ |  |

1. **Laboratory result report form**

**Unique code number: ________ Patient Card number: ________**

**Time of sample received:** _____________

| **Parameter** | **Description** | | | |  |
| --- | --- | --- | --- | --- | --- |
| Stool sample consistency | 1. Formed 2. Lose or Watery 3. Blood stained 4. Mucoid 5. Parasite 6. Other ____ | | | |  |
| Stool color | ___________ | | | |  |
| Pathogen detected | 1. ________________ 2. ________________ 3. ________________ 4. None | | | |  |
| Drug susceptibility profiles (for the identified enteric bacterial pathogens) | **Name of the antibiotic** | **Sensitivity pattern** | | | |
|  |  | **S** | **IS** | **R** |  |
|  | 1. SXT |  |  |  |  |
|  | 1. Amp 2. …, etc |  |  |  |  |
| Name and signature of the investigator | ____________________ |  |  |  |  |
| Date and time of report | **____________________** |  |  |  |  |
